# Myelomatous pleural effusion

**DOI:** 10.1002/jha2.446

**Published:** 2022-05-25

**Authors:** Silvia Escribano Serrat, Javier Cucharero Martín, Fiorella Medina Salazar, Estefanía Bolaños Calderón, Isabel Ortega Madueño, Celina Benavente Cuesta, Fernando Ataúlfo González Fernández

**Affiliations:** ^1^ Hematology and Hemotherapy Department Hospital Clínico San Carlos, IdISSC Madrid Spain; ^2^ Clinical Analysis Department Hospital Clínico San Carlos Madrid Spain

**Keywords:** infiltration, multiple myeloma, myelomatous pleural effusion, plasma cells, pleural effusion

1

A 66‐year‐old woman was admitted to the emergency department reporting 1 week of asthenia and fever that had persisted despite antibiotherapy. She had been diagnosed of smoldering multiple myeloma (MM) IgG kappa 11 years before. However, treatment with bortezomib, lenalidomide and dexamethasone (VRd) was not started until the previous year, when the patient presented with rib pain and the diagnostic workup was repeated. Bone marrow aspirate showed infiltration of clonal plasma cells, and bone disease was demonstrated in positron emission tomography/computed tomography scan. After the fourth cycle of VRd, an increase in serum monoclonal component was observed.

Urgent laboratory tests showed hemoglobin, white blood cell and platelet counts of 110 g/L, 7.5 × 10^9^/L and 190 × 10^9^/L, respectively, with 1570 mg/dl of IgG, 643 mg/dl of kappa light chain, 933 U/L of LDH, and 6.5 mg/L of beta2‐microglobulin. Chest X‐ray revealed left pleural effusion (PE). A thoracocenthesis was performed. Cytological examination of PE and peripheral blood smear showed plasma cell infiltration (Figure 1A–F). PE flow cytometry revealed 96% infiltration of abnormal cells expressing CD38, CD138, CD56^dim^ and CD19 loss expression (Figure 2A–E). Pleural fluid electrophoresis and immunofixation revealed monoclonal IgG kappa (Figure 2F–G) and bone marrow aspirate showed 90% infiltration of plasma cells. Blood and bone marrow cultures were negative. A diagnosis of myelomatous pleural effusion (MPE) was made. Treatment with daratumumab, carfilzomib and dexamethasone was started. However, 10 days later the patient died.

**FIGURE 1:**
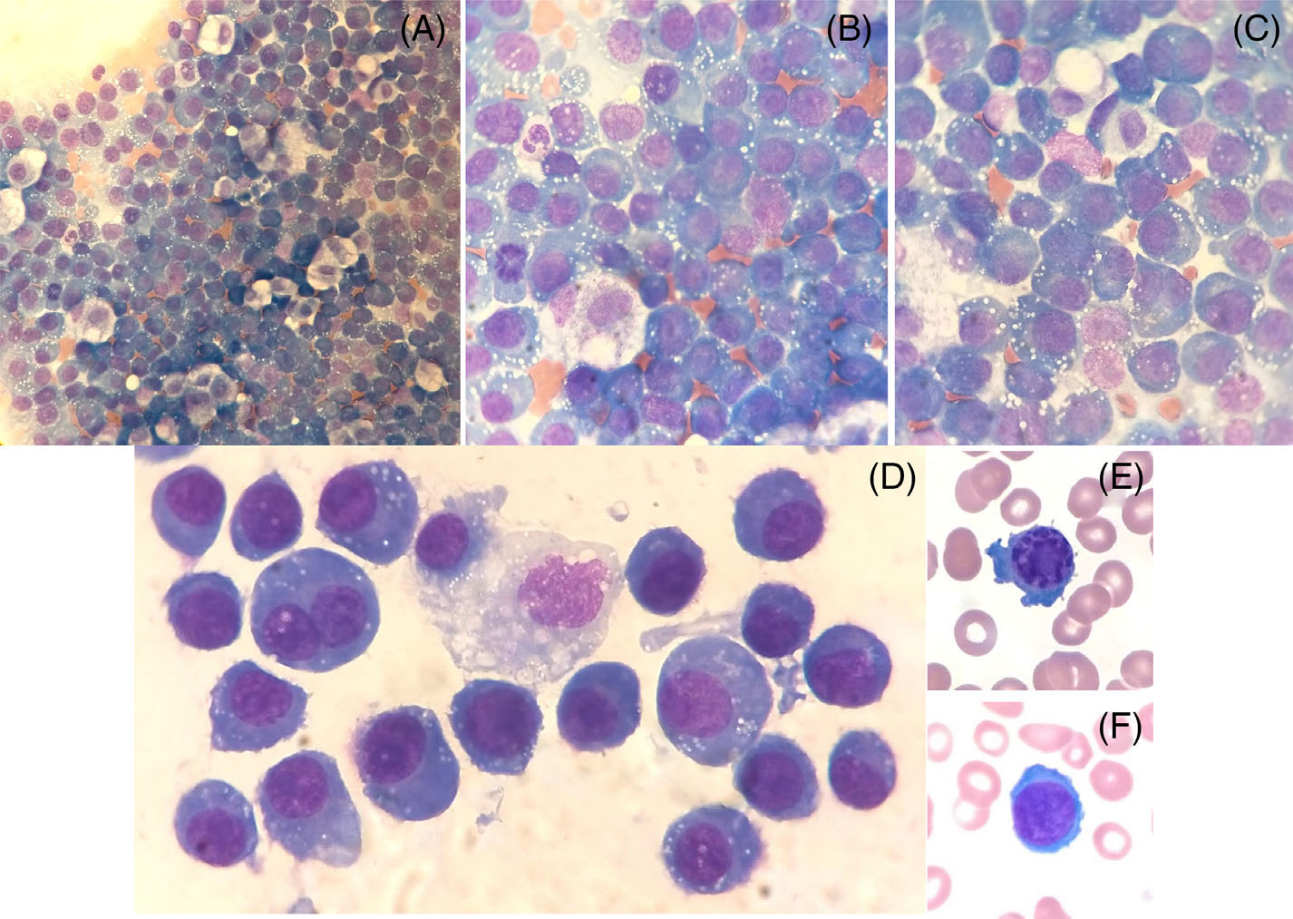
(A) Wright–Giemsa stain, 400× objective. (B–F) Wright–Giemsa stain, 1000× objective. (A–D) Pleural effusion microscopic findings. (E–F) Plasma cells in peripheral blood smear

**FIGURE 2:**
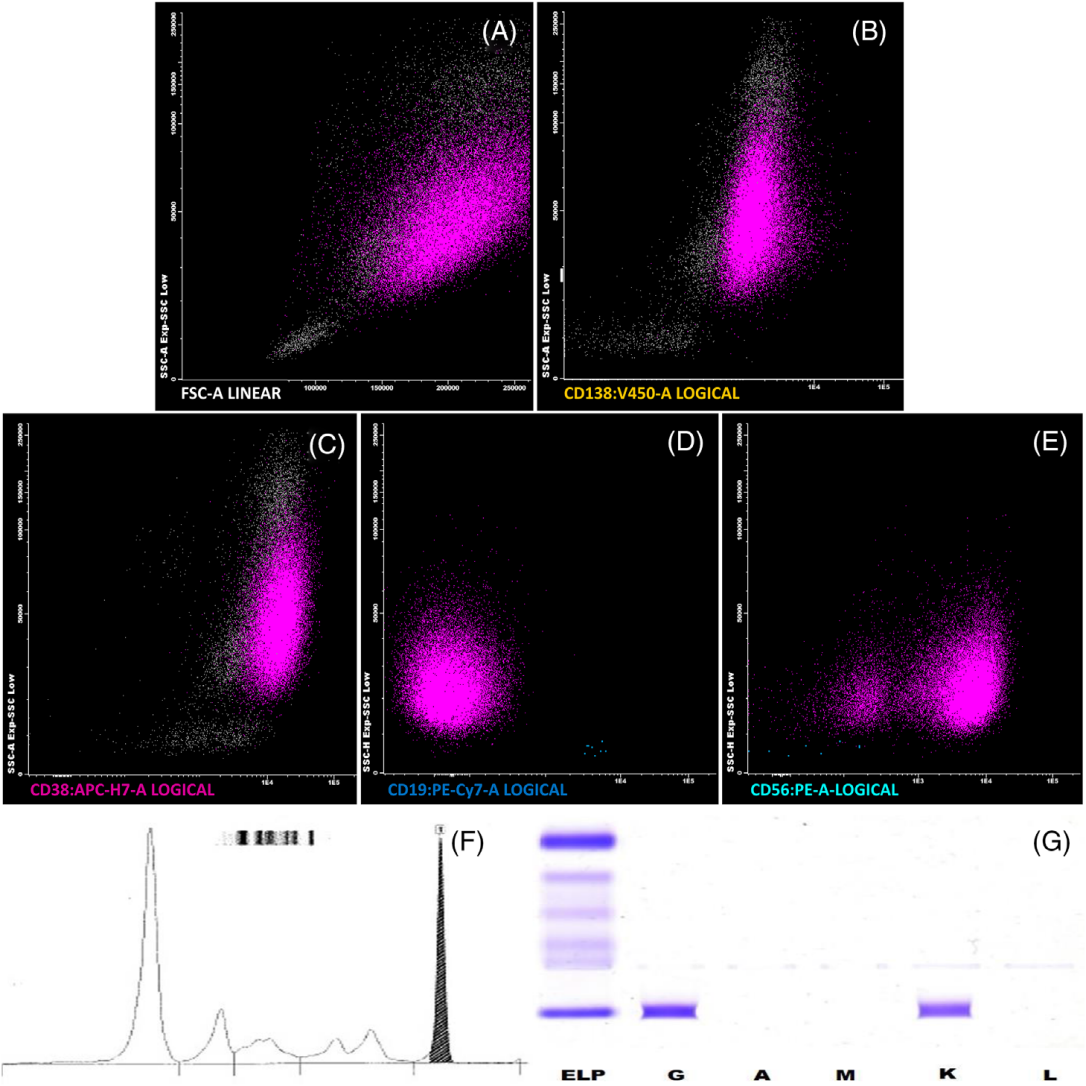
(A–E) Pleural effusion flow cytometry. (F–G) Pleural effusion electrophoresis and immunofixation

MM is a malignant plasma cell disorder that accounts for approximately 10% of all hematologic malignancies. Only 6% of these patients develop pleural effusion, mostly benign [1]. MPE is a very rare finding, present in less than 1% of all cases. Cytological identification of malignant plasma cells within pleural effusion is considered the most accurate diagnostic method. There is no standardized therapy. The prognosis is dismal, with an overall median survival ranging from 2 to 4 months [2, 3, 4].

## CONFLICT OF INTEREST

The authors declare no conflict of interest.

## ETHICS STATEMENT

All procedures performed in studies involving human participants were in accordance with the ethical standards of the institutional and/or national research committee and with the 1964 Helsinki declaration and its later amendments or comparable ethical standards.

## PATIENT CONSENT STATEMENT

Informed consent was obtained from a first degree relative of the patient included in the study.

## AUTHOR CONTRIBUTIONS

All authors contributed to the study conception and design. Material preparation, data collection and analysis were performed by Silvia Escribano Serrat, Javier Cucharero Martín, Fiorella Medina Salazar, Estefanía Bolaños Calderón, Isabel Ortega Madueño and Fernando Ataúlfo González Fernández. The first draft of the manuscript was written by Silvia Escribano Serrat and all authors commented on previous versions of the manuscript. All authors read and approved the final manuscript.

## FUNDING INFORMATION

None.
